# Evaluation of the physicochemical parameters of edible oils sold in the three cities of Burkina Faso

**DOI:** 10.1002/fsn3.2819

**Published:** 2022-03-14

**Authors:** Kabakdé Kaboré, Kiéssoun Konaté, Hemayoro Sama, Roger Dakuyo, Abdoudramane Sanou, David Bazié, Mamounata Diao, Mamoudou Hama Dicko

**Affiliations:** ^1^ Laboratory of Biochemistry, Biotechnology, Food Technology and Nutrition University Joseph Ki‐ZERBO Ouagadougou Burkina Faso; ^2^ Laboratory of Biochemistry and Applied Chemistry University Joseph Ki‐ZERBO Ouagadougou Burkina Faso

**Keywords:** edible oils, physicochemical, refined oils, unrefined oil

## Abstract

The edible oil needs of African countries are met by imported or locally produced ones. Therefore, consumers are generally confronted with a choice of edible oils of poorly controlled quality. However, quality control of edible oils for local consumption is of high necessity. This study aimed to assess the quality of edible oils sold and consumed in some cities in Burkina Faso. Oil samples collected in the cities of Dédougou, Koudougou, and Nouna were used for several analyses. Oil samples from palm, refined and unrefined cottonseed, and groundnut were collected. Standard methods were used to assess the physicochemical quality parameters of the oils, including the peroxide value, water and volatile matter content, acid value, traces of soap, and mineral oil contents. The parameters varied significantly depending on the oil type, but not by the locality of origin. The peroxide indices had varied from 3.24 to 39.99 mEq O_2_/kg oil. The acid indices varied from 0.22 mg KOH/g to 1.24 mg KOH/g. The water and volatile matter contents ranged from 0.04% to 0.88%. The test for traces of soap gave values ranging from 0 to 76 ppm. For the mineral oil test, four samples of cottonseed oil collected in Dedougou gave positive results. Compared to international reference standards, in particular the Codex Alimentarius standard, it may constitute a health risk for consumers. The poor storage, distribution, and marketing conditions of the oils could explain their poor quality. In order to provide consumers with quality oils, regular controls must be undertaken in the places where the oils are stored and/or marketed.

## Graphical abstract

Oil is an important food for the human organism; however, the oils sold on our markets do not all respect the quality standards. In Burkina Faso, mainly in Koudougou, Dédougou, and Nouna, high levels of mineral oils and traces of soap have been detected, in addition to extensive oxidation, and these oils can therefore constitute a public health problem. Political leaders should therefore ensure that the quality standards for all oils sold on their markets are strictly respected.

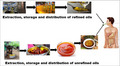

## 1 INTRODUCTION

Edible oils play an essential role in our diet. In addition to providing a nutritional function, they contribute to energy supply and are sources of essential fatty acids particularly linoleic and alpha‐linolenic acids (Okparanta et al., 2018). They also participate in the supply and transport of fat‐soluble vitamins including A (in terms of carotenoids), D, E, K, and other constituents of nutritional interest such as phytosterols or phenolic compounds especially for vegetable oils (Mengistie et al., 2018). This high nutritional potential of oils is due to their stability (Benmohamed et al., 2020).

In Burkina Faso, vegetable edible oils are essentially made up of locally produced oils and imported ones. During 2009–2010, the demand for oil on the Burkinabe market was in the order of 60,000–65,000 tons, while the annual production capacity of processing units was estimated at 20,000 tons. This production has increased to reach 86,000 tons in 2014 with an estimated demand between 100,000 and 120,000 tons per year (Hougni & Vognan, 2021; USAID, 2017). These oils are produced by several enterprises including industrialized, semi‐industrialized, and informal or artisanal ones. Although not included in the list of regulated oil mills, artisanal oil mills contribute significantly to national oil production (Guissou & Ilboudo, 2013). Nearly 6%–10% of consumed oil in Burkina is from artisanal units (Song‐Naba, 2016). The origin and conditions of production, storage, and marketing of edible oils raise many concerns about their quality (Ahouansou & Agbobatinkpo, 2018) (Zio et al., 2020). The quality control processes of edible oils are important parameters because they determine the quality of the shelf life and thus the economic value of the oils (Macarthur et al., 2021). Alteration of edible oils such as rancidity can pose health risks, including cardiovascular disease, cancer, etc., due to the occurrence of toxic and reactive oxidation products ((Huang & Ahn, 2019; Wu & Zhou, 2021). Potential toxicity is also suspected from certain compounds of oxidative degradation of unsaturated fatty acids, including cyclic monomers, polymers, and furans, especially in the case of oxidative degradation associated with thermal degradation and rancid oils (Konuskan et al., 2018). Rancid oil forms harmful free radicals in the body, which could cause cell damage and have been linked to type‐II diabetes, Alzheimer's disease, and other metabolic disorders. Rancid oils can also cause digestive problems and can deplete the body of B group and E vitamins (Okparanta et al., 2018). Thus, to ensure the quality of the oils local people consume, evaluation of their physicochemical parameters is necessary. Unfortunately, in Africa, particularly in Burkina Faso, very few studies have been carried out on edible oils. Thus, the objective of this study is to evaluate the physicochemical parameters of edible oils sold in the three cities of Burkina Faso.

## 2 MATERIALS AND METHODS

### 2.1 Study framework

The National Public Health Laboratory (LNSP) is a non‐hospital public health establishment, created by decree n°91,654 of October 09, 1991 and officially opened its doors on November 15, 2002. The LNSP is located on the circular boulevard Tangsoba. Its main objective is to be a reference for biomedical, toxicological, physicochemical and microbiological analyses, sanitary quality controls and expertise related to medical biology, food, nutrition, pharmacy, water, environment and any field related to public health and sanitary safety. It includes technical departments supported by an administrative organization common to the public health institutions of Burkina Faso. For decentralized coverage of the national territory, the institution has two regional directorates: in Bobo‐Dioulasso and in Ouagadougou.

### 2.2 Brief presentation of the collection zones and biological materials

The city of Koudougou has approximately 183,332 inhabitants, Dedougou has 118,727 inhabitants, and Nouna has 95,599 inhabitants. Samples (60) were collected in the above three cities (20 samples/city). The distribution of the samples in Koudougou is as follows: 2 of groundnut oil, of 2 unrefined palm oil, and 16 of refined palm oil; the in Dédougou: 12 of refined palm oil and 08 of refined cottonseed oil; in Nouna: 7 of refined cottonseed oil, 9 of refined palm oil, 2 of groundnut oil, and 2 of unrefined palm oil.

## 3 METHODS

### 3.1 Peroxide value measurement

Peroxide value is a measure of peroxides contained in the oil. It is determined by measuring iodine released from KI. A known measured weight of oil samples is dissolved in acetic acid then chloroform and saturated KI mixture are added to the sample and the amount of iodine liberated from KI by the oxidative action of peroxides present in the oil is determined by titration with standard sodium thiosulphate using starch solution as an indicator (AOAC, 1984).

### 3.2 Determination of the moisture and the volatile matters content

The moisture and the volatile matters content is according to standard (ISO, 2016).

### 3.3 Determination of the acid value

The fat matters alter themselves by aging while giving birth by hydrolysis to free fatty acids. The acidic value of an oil is determined by titration of the free fatty acids with the help of an ethanolic solution of potassium hydroxide (ISO 660, 2020).

### 3.4 Determination of the soap traces

It is defined as the content of the soap in the oil that is soluble in the acetone with 3 to 4% of water expressed in part by million (ppm): it informs on the efficiency of the separators.

It consisted of titlesoaps freed in the acetone, in presence of a colorful indicator (blue of bromophenol), in a solution of 0.01 N HCl (AOCS, 2009).

### 3.5 Statistical analysis

Tukey's test, descriptive statistics, analysis of variances (ANOVA), and principal component analysis were established using Excel software; GraphPad, and XLSAT version 2018.

## 4 RESULTS AND DISCUSSION

### 4.1 Results

#### 4.1.1 Mineral oil

It appeared that four samples of cotton refined oil from Dédougou contained mineral oils. The samples of the other localities did not contain any mineral oils.

Samples displayed different levels of peroxides, acids, and soap traces in the cities of Koudougou, Dédougou, and Nouna (Figures 1, 2, 3, 4, 5, 6, 7, 8, 9, 10, 11, 12).

**FIGURE 1:**
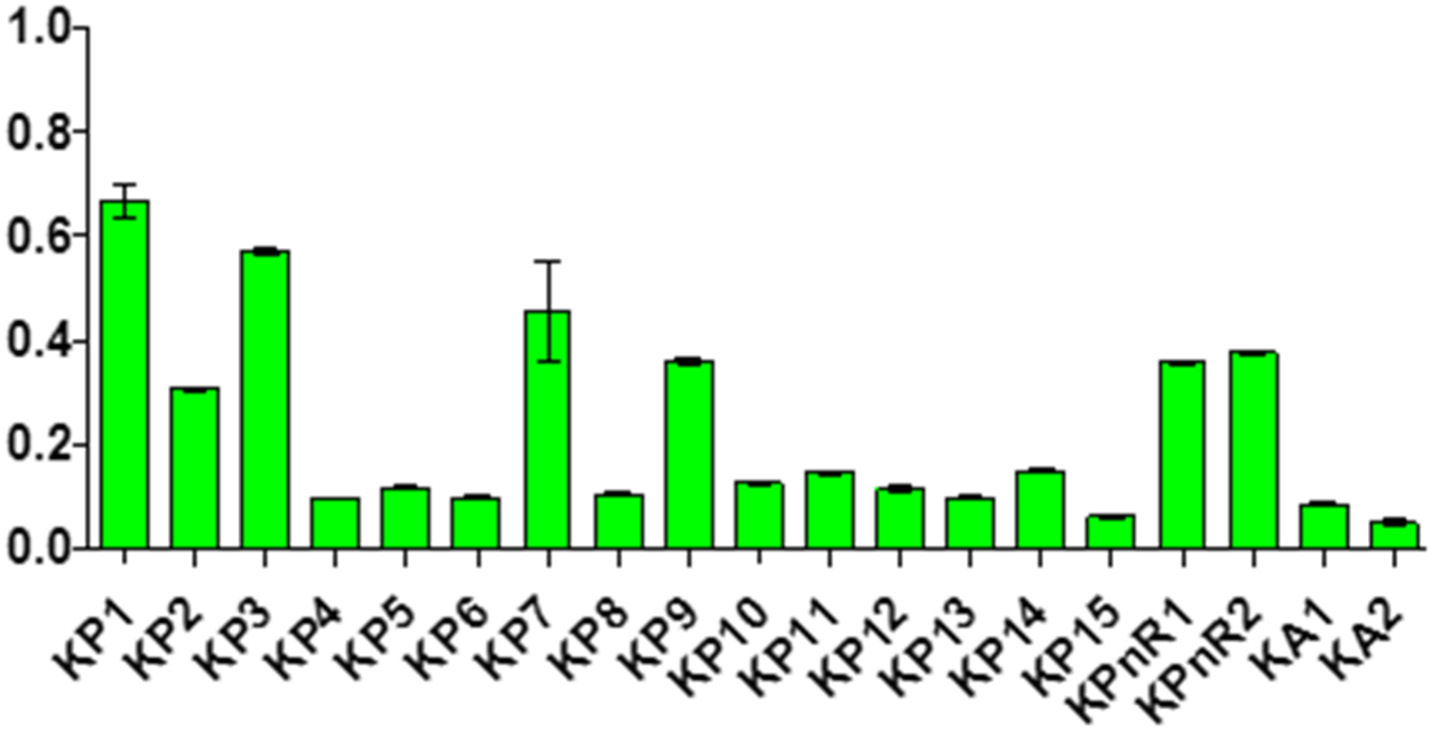
Evaluation of the moisture and the matters content of samples collected in Koudougou

**FIGURE 2:**
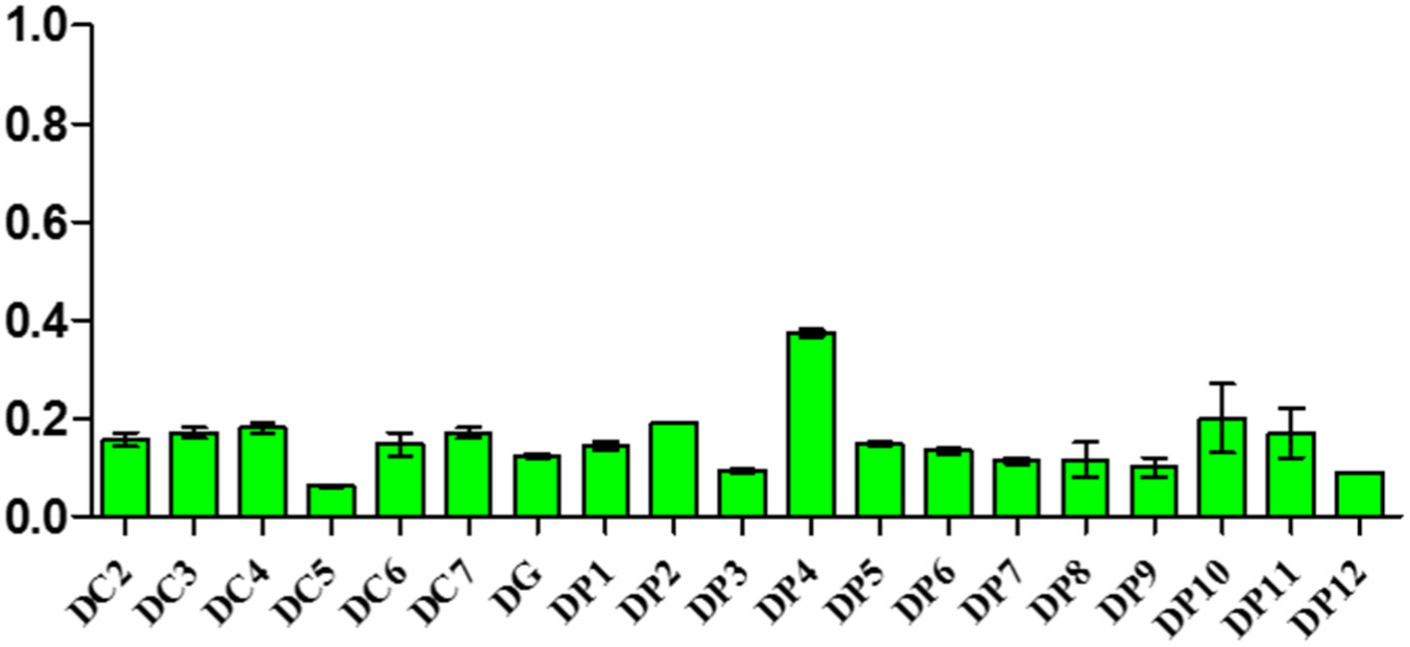
Evaluation of the moisture and the matters content of samples collected in Dédougou

**FIGURE 3:**
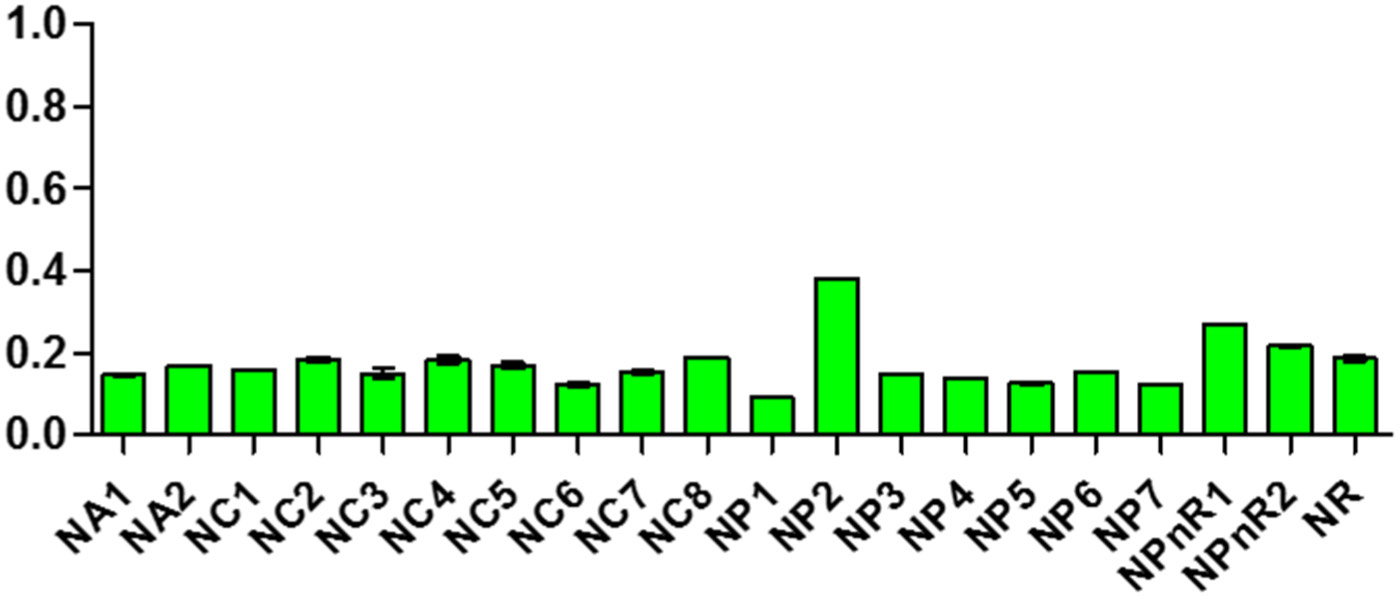
Evaluation of the moisture and the matters content of samples collected in Nouna

**FIGURE 4:**
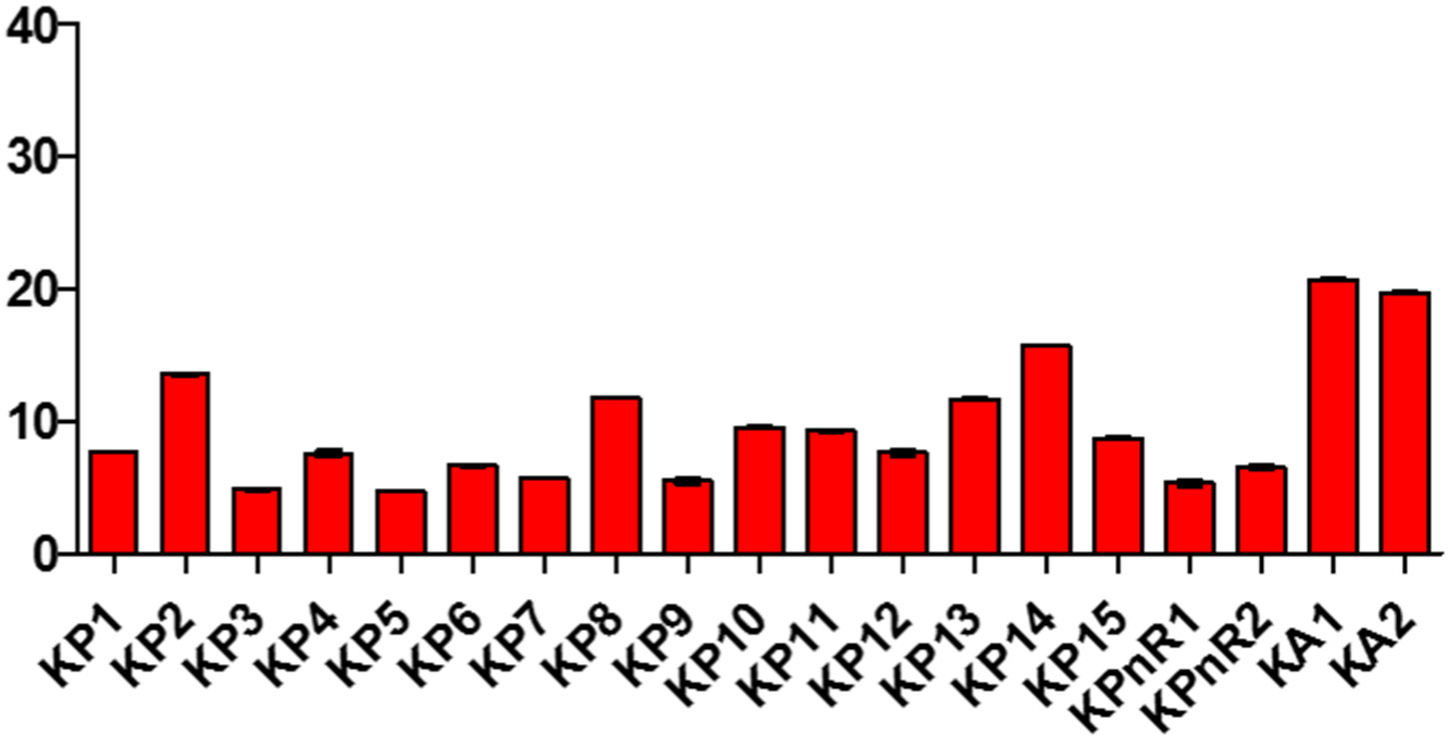
Evaluation of peroxide value of samples collected in Koudougou

**FIGURE 5:**
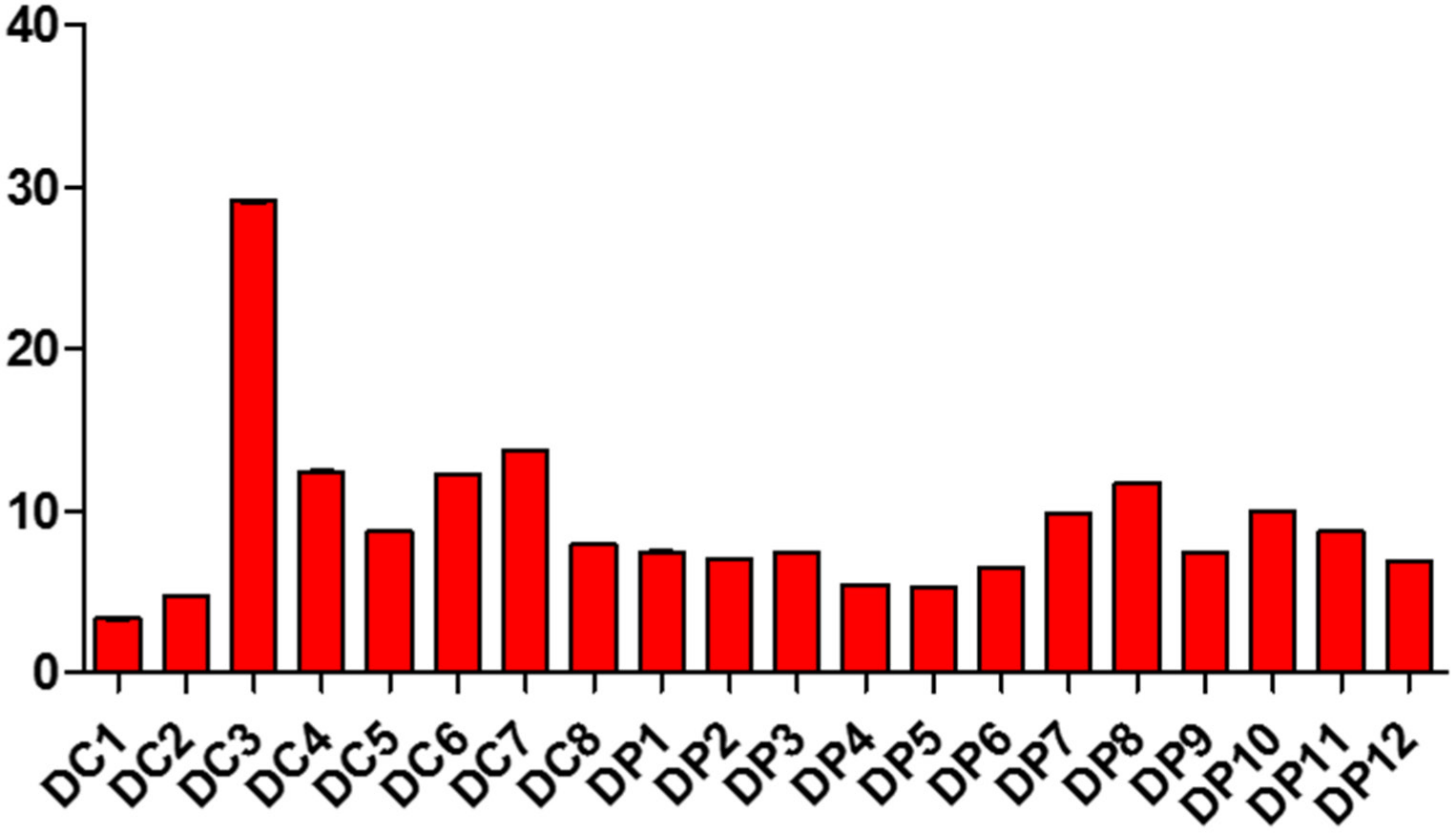
Evaluation of peroxide value of samples collected in Dédougou

**FIGURE 6:**
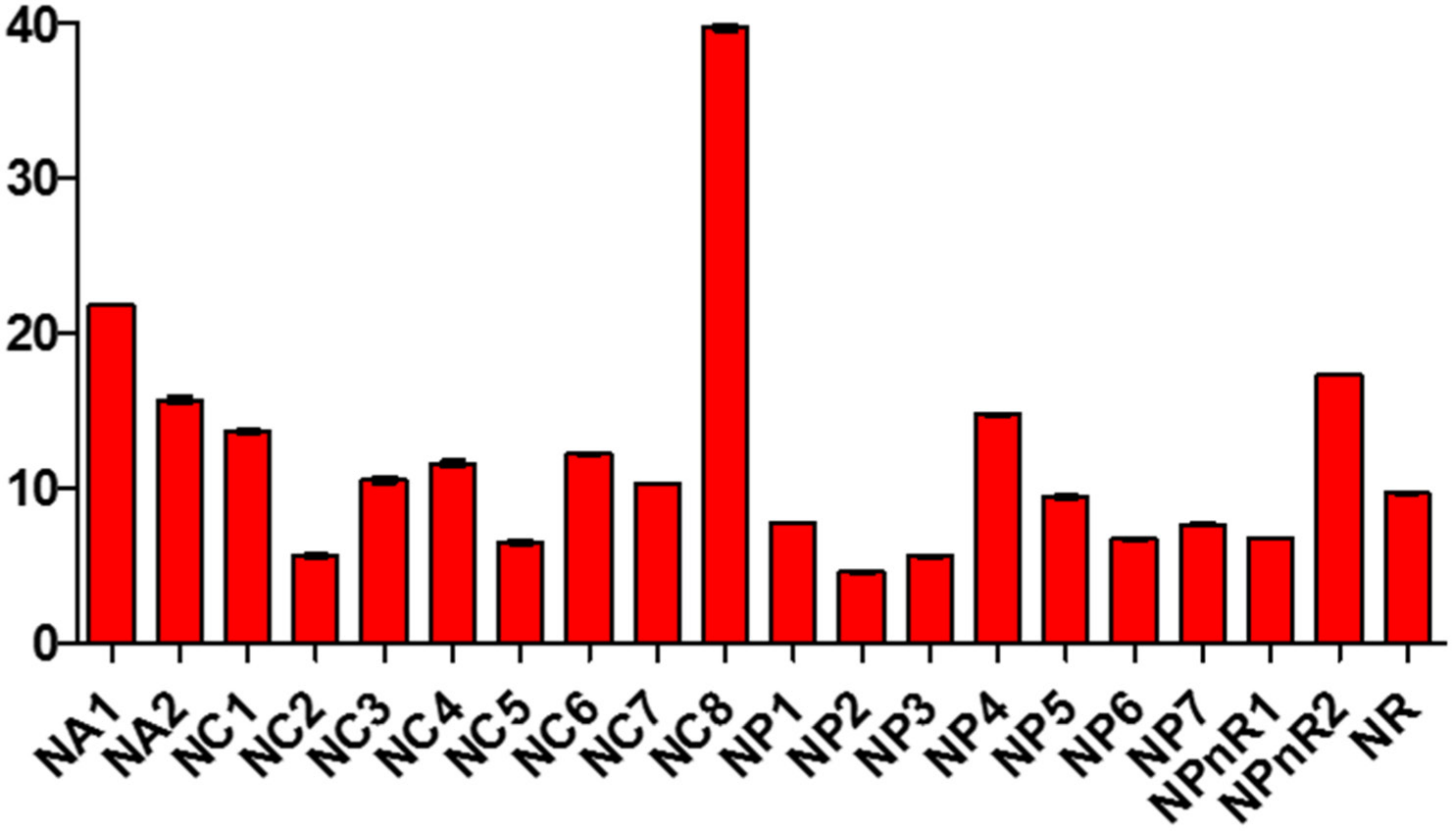
Evaluation of peroxide value of samples collected in Nouna

**FIGURE 7:**
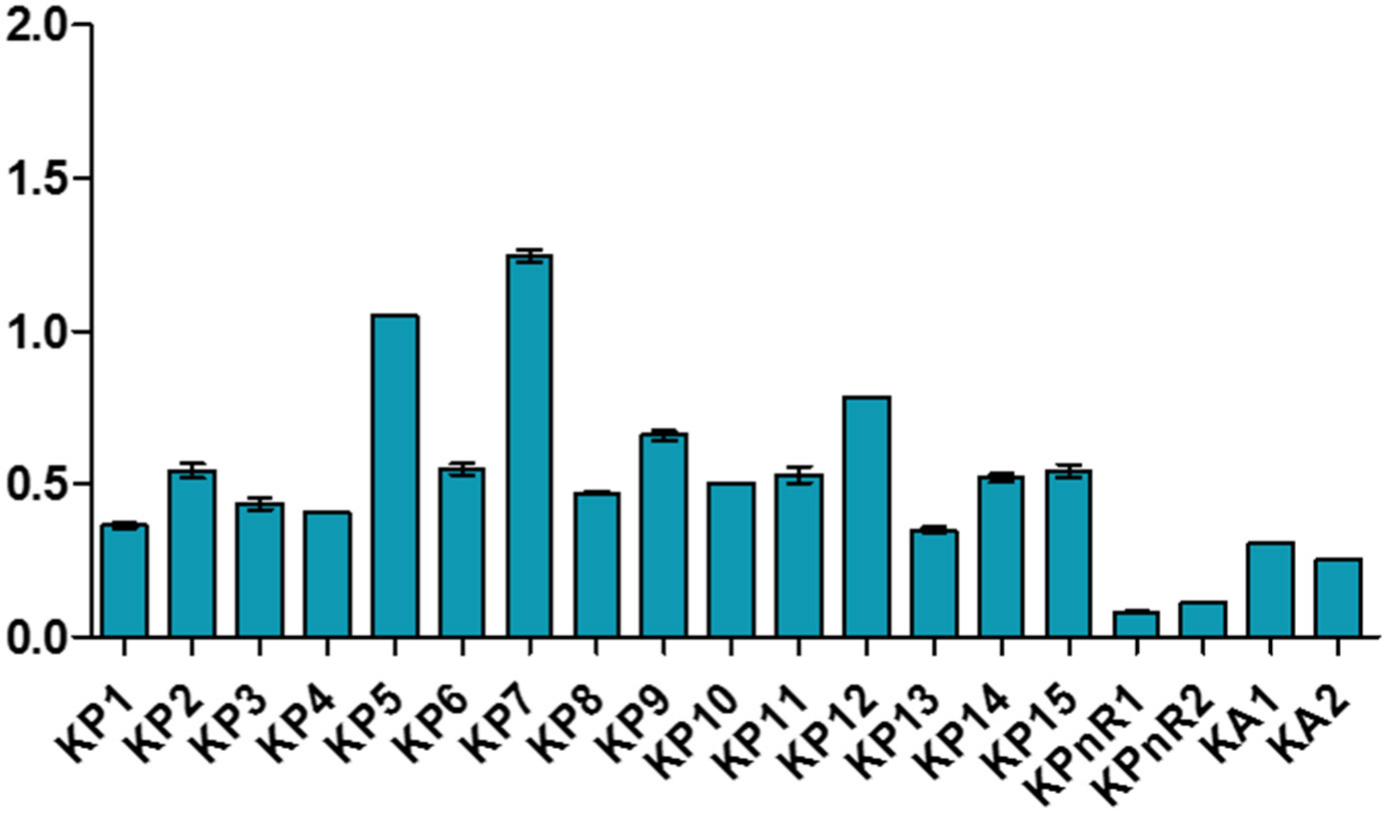
Evaluation of acid indices of samples collected in Koudougou

**FIGURE 8:**
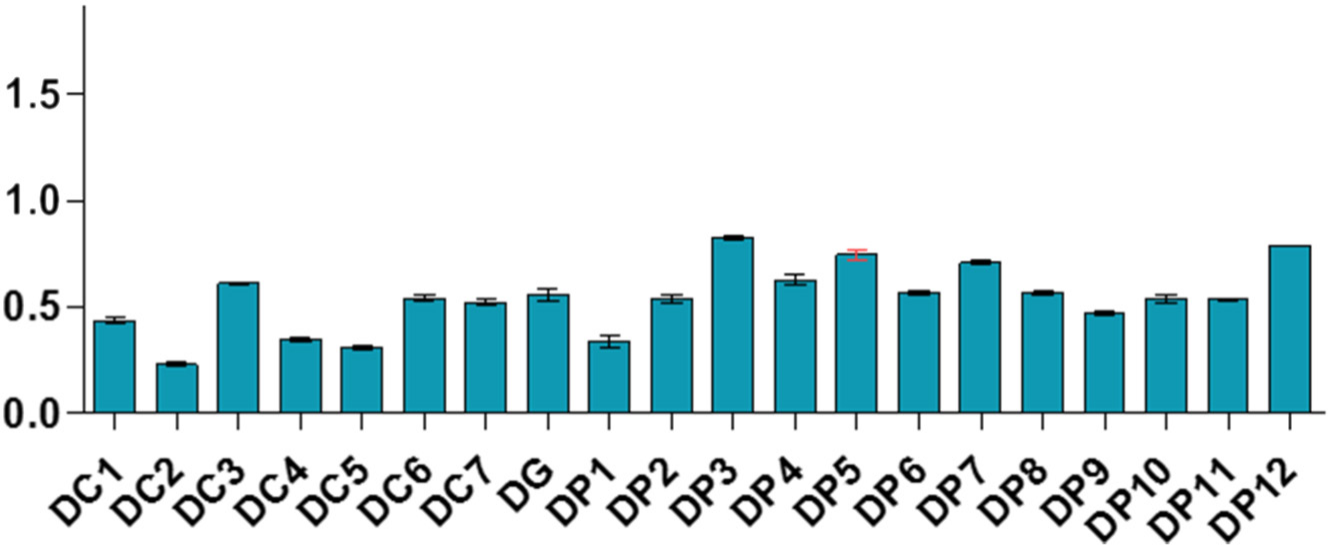
Evaluation of acid indices of samples collected in Dédougou

**FIGURE 9:**
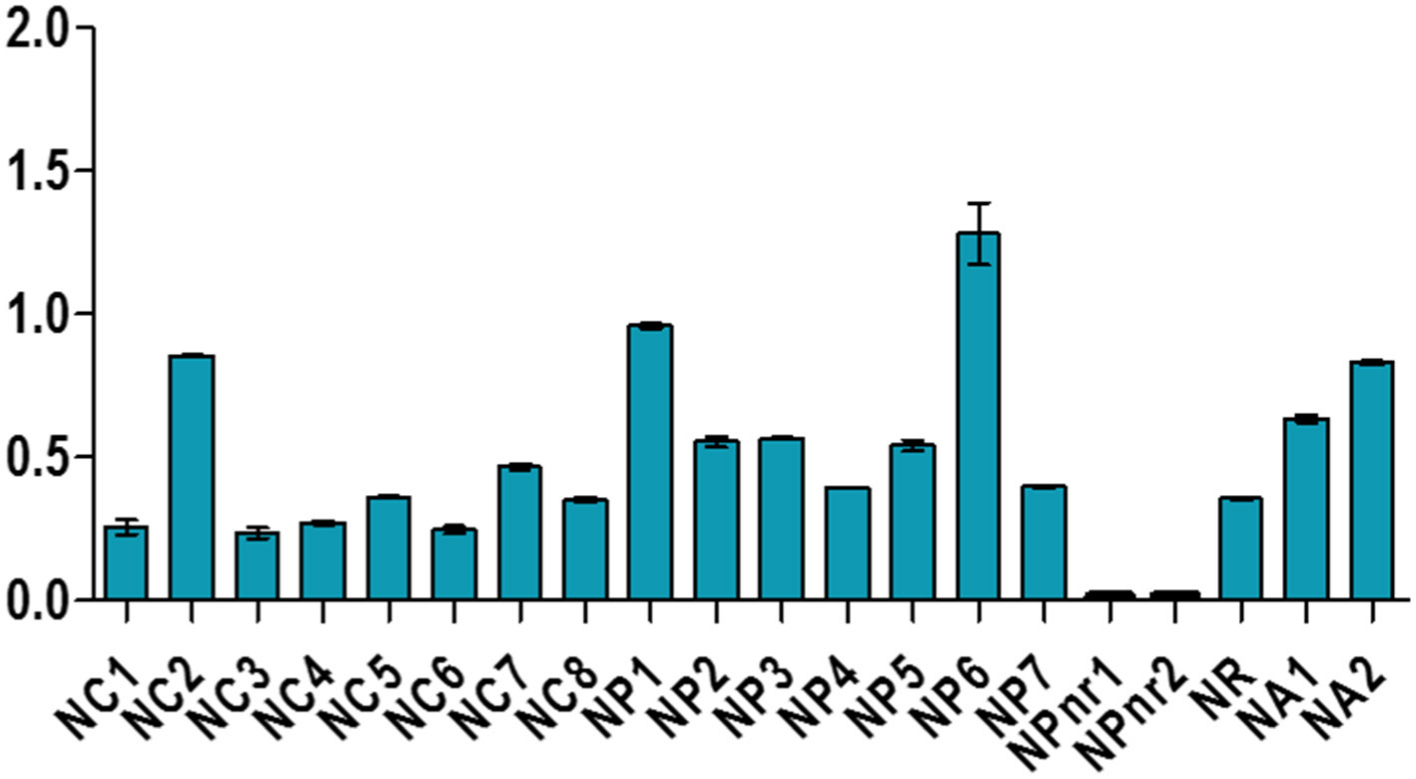
Evaluation of acid indices of samples collected in Nouna

**FIGURE 10:**
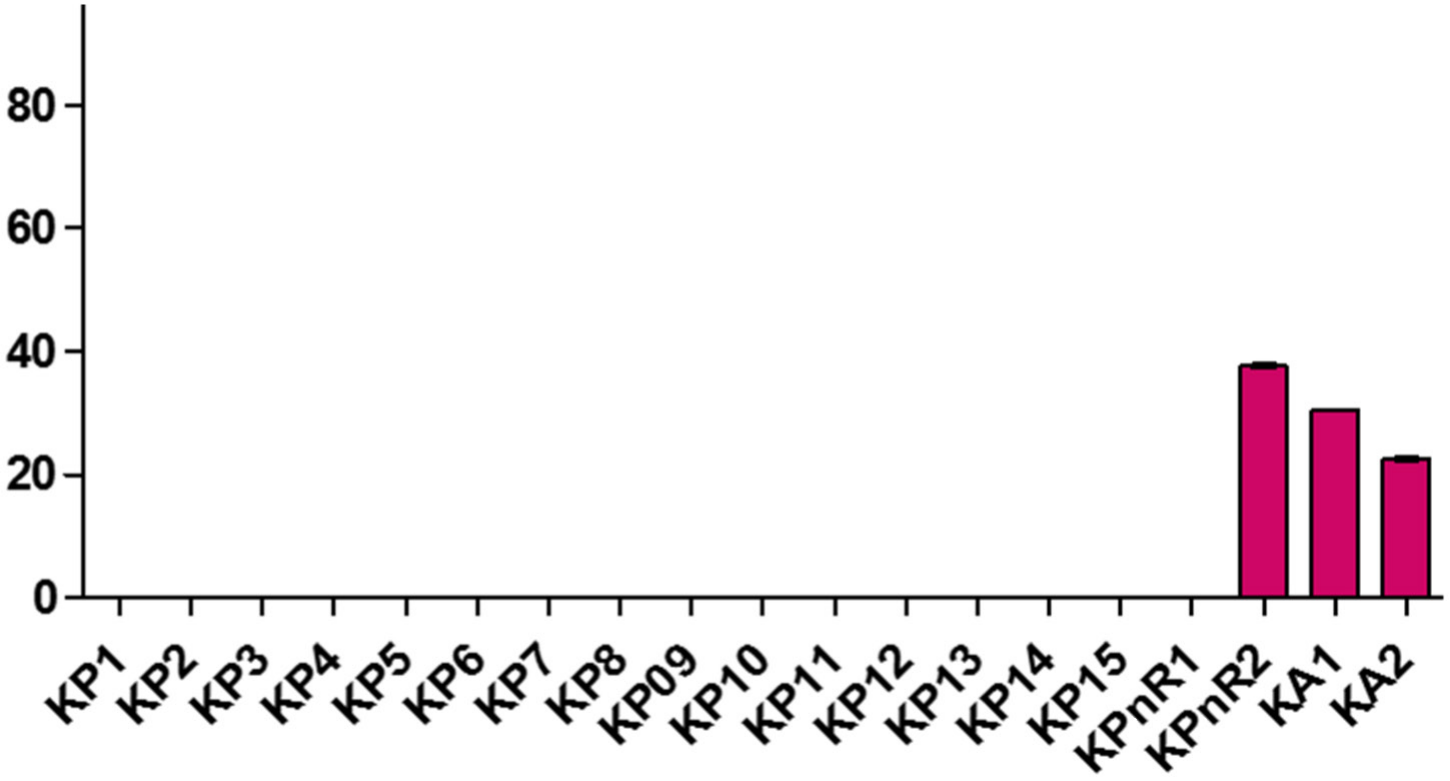
Evaluation of traces of soap of samples collected in Koudougou

**FIGURE 11:**
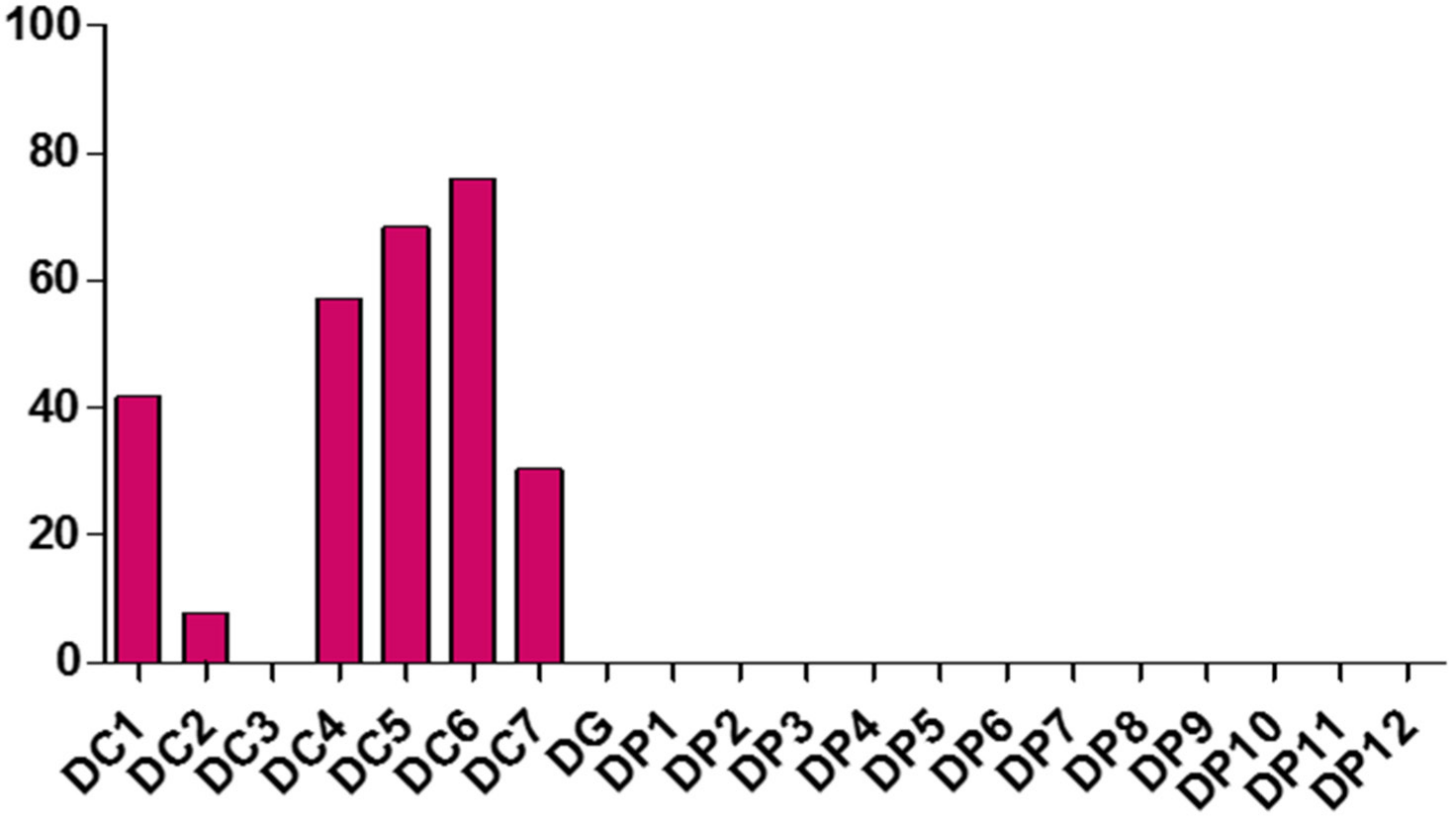
Evaluation of traces of soap of samples collected in Dédougou

**FIGURE 12:**
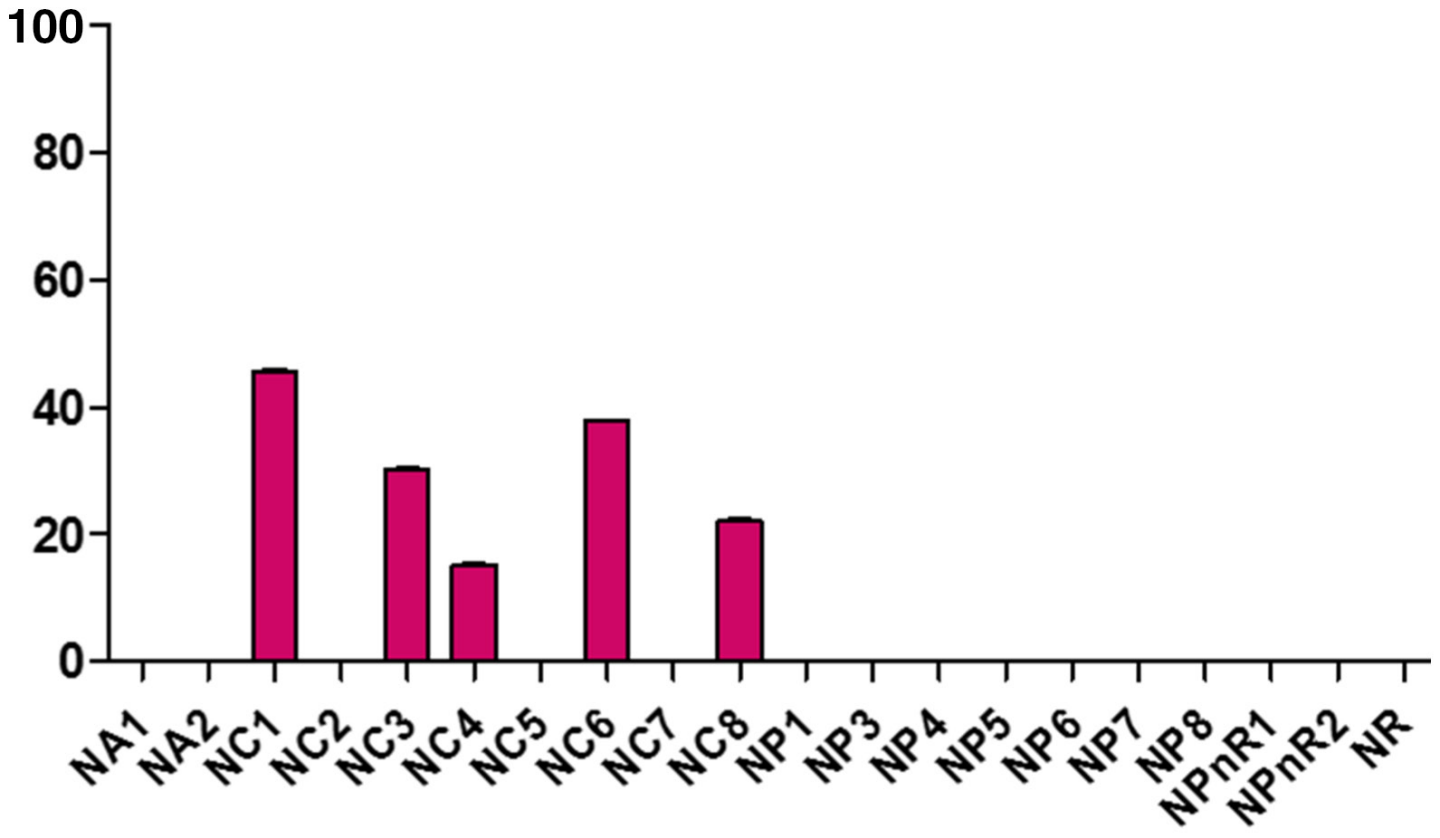
Evaluation of traces of soap of samples collected in Nouna

### 4.2 Discussion

**FIGURE 13:**
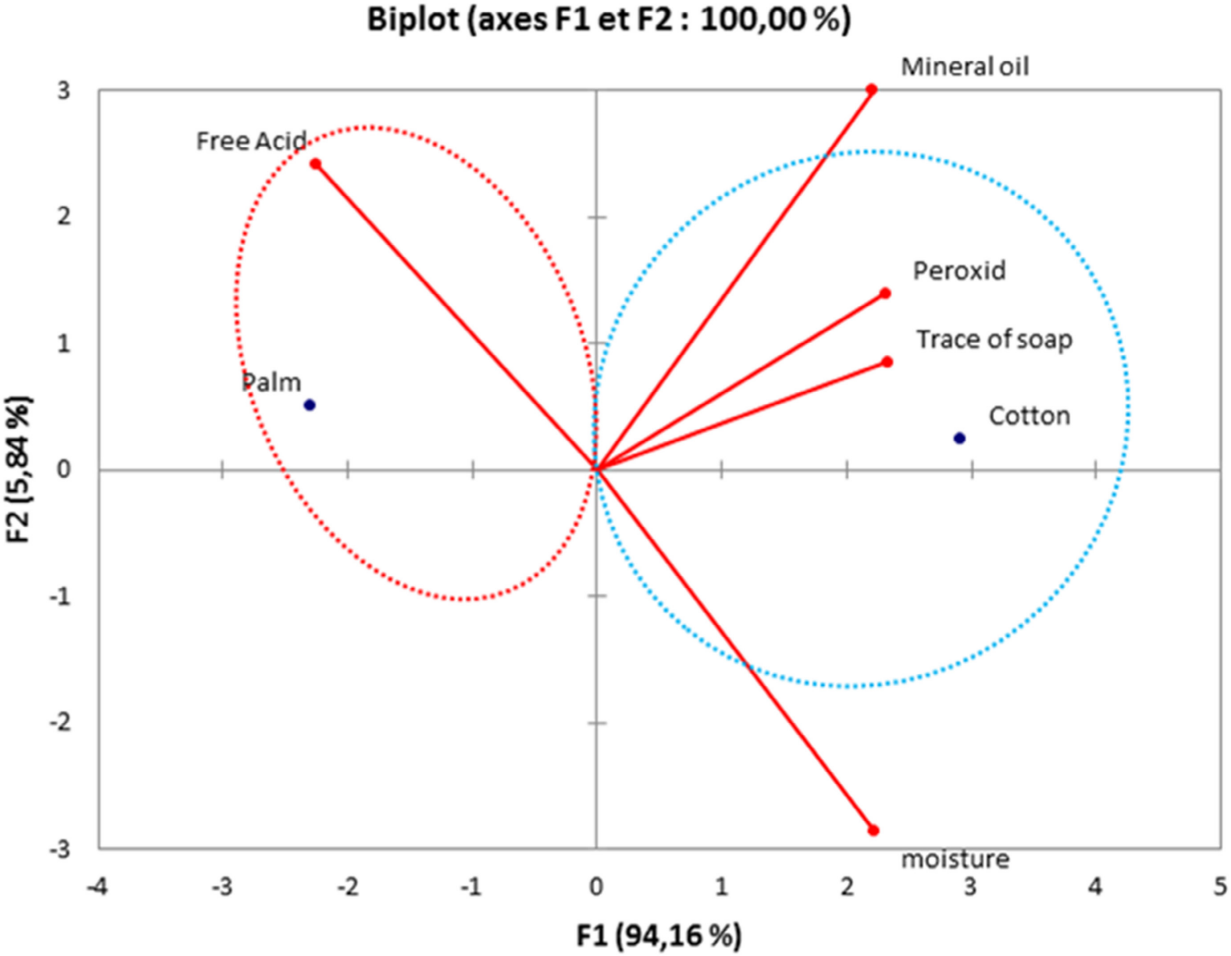
Principal components analysis

The moisture and volatile matter contents of samples ranged from 0.04% to 0.88% ±0.22. Among samples, 35% from Koudougou, 25% from Dédougou, and 15% from Nouna displayed values higher than the maximum value for moisture and volatile matter content set by the Codex Alimentarius and those reported elsewhere (Hasan et al., 2018); but they are similar to data found by Odoh et al. (2017). These high levels could be related to the extraction processes and could influence the conservation of the oil by promoting the hydrolysis of free fatty acids and their oxidation (Odoh et al., 2017). Indeed, oil undergoes more significant alteration during storage such as acidification, oxidation, and is conducive to the development of microorganisms (Macarthur et al., 2021) (Figure 13).

The peroxide value of the refined oils varies from 3.24 to 39 mEqO_2_/kg with averages of 8.52 O_2_/kg mEq, 9.31 O_2_/kg mEq, and 10.92 O_2_/kg mEq for the Koudougou, Dédougou, and Nouna samples. These data are similar to those found by Almeida et al. (2019). However, 30% of samples showed values above 10 mEqO_2_/Kg, the maximum value set by the Codex Alimentarius for refined oils, which would indicate advanced oxidation for these samples. It is known that oxidative deterioration of oils occurs much faster when stored at high temperature and exposed to light than under other storage conditions (Almeida et al., 2019).

For unrefined oils, peroxide values ranged from 4.99 to 21.97 mEqO_2_/kg for the Koudougou and Nouna oil samples, respectively. Five percent of the unrefined oil samples showed values above the maximum level set by the Codex Alimentarius (15 mEq O_2_/kg). Nevertheless, these findings are different from those found elsewhere (Chiboret al., 2018; Tańska et al., 2016). According to these analyses, high temperature, visible light, and oxygen can easily increase the peroxide value of oils. Oils with a peroxide value greater than 15 meqO_2_/kg may be dangerous to health by increasing reactive oxygen species as well as secondary products of lipid peroxidation, which are sources of cardiovascular and inflammatory diseases (Konuskan et al., 2018).

For the acid indices of refined oil samples, they ranged from 0.22 to 1.24 mg KOH/g with respective averages of 0.58 mgKOH/g, 0.53 mgKOH/g, and 0.5 mgKOH/g for Koudougou, Dédougou, and Nouna, respectively. For unrefined oil samples, the value ranged from 0.08 mgKOH/g to 0.84 mgKOH/g. This may be due to the presence of endogenous enzymes and antioxidants that prevent the hydrolysis of fatty acids (Benguendouz et al., 2017). Of all refined oil samples, 20% had acidity values greater than 0.6 mg KOH/g, the maximum value set by the Codex Alimentarius; while for unrefined oils these acidity values are lower than the maximum value, 4 mgKOH/g, set by the Codex Alimentarius for virgin oils. The low acid value of unrefined oils reveals their quality in contrast to the 20% of refined oil samples, which have values above the maximum values of Codex Alimentarius. According to Bettahar et al. (2016), the acid indication is a function of free fatty acids and characterizes the state of change of the oil by hydrolysis. They show that pure oil has low acidity. High acidity indicates that the oil may be oxidized and contains free fatty acids formed during extraction and or storage (Bettahar et al., 2016).

The refined oils sampled in Koudougou were free of soap. One unrefined palm oil sample had traces of soap of 37.99 ppm and two peanut oil samples contained traces of soap of 22.79 ppm and 30.39 ppm, respectively. For the Nouna samples, 30% of the cotton samples contained traces of soap with values ranging from 15.2 ppm to 38 ppm. All of these values are below the Codex Alimentarius maximum value of 50 ppm. As for the Dédougou samples, with traces of soap values ranging from 7.6 ppm to 76 ppm, 15% of the cottonseed oil samples had traces of soap values higher than the maximum value of the Codex Alimentarius. These high values of soap traces could be justified by the lack of control of oil refining processes. Furthermore, the presence of soap in food oils can be explained by a chemical deacidification operation (Motri et al., 2017).

According to Kharroubi Mariem and Bellali Fatima (2017), chemical neutralization has been widely applied in the industry to remove free fatty acids in vegetable oils. This results in a loss that is increased by the binding of the oil to the soap formed (Mariem & Fatima, 2017). The high soap content in samples DC4, DC5, and DC6 is due to the non‐control of the refining process or the use of inadequate materials. These oils should be removed from the consumer market, as they may pose a health risk to the consumer. The presence of trace amounts of soap in peanut oils may be due to contamination with the use of recycled soap‐washed containers.

As for the search for mineral oils, we found that four cotton samples taken in Dédougou show a positive result. These results are similar to those obtained by Ahmad et al. (2019) who have worked on mineral oil saturated hydrocarbons in crude palm oils from Malaysia mill. Mineral oils suspected of being carcinogenic can migrate to food through different sources. Indeed, Gharbi et al. (2016) showed that the transportation of crude oil presents a risk of possible contamination by mineral oils (Gharbi et al., 2016). Li et al. (2017) demonstrated that mineral oils could be encountered in all oils following several modes of contamination. They showed the presence of mineral oils in 23 samples of which three samples were contaminated with more than 50 mg/kg of MOSH and the highest level of contamination was found in one of the rice oils, in which the MOSH concentration reached 713.36 mg/kg. According to this author, it is necessary to systematically detect mineral oil contamination in vegetable oils for food safety (Li et al., 2017).

The principal component analysis of these oils reveals two groups according to the parameters studied. A first group showing palm oil strongly correlated with the acid value. This high content of free fatty acids is may be due to the storage time of the seeds which negatively influences the acidity of the oil (Nanda et al., 2020). In addition, the increase in acidity is partly due to microbial lipase activity and self‐hydrolysis of the oil (Domonhédo et al., 2018). Also, there is a positive correlation between acidity content and moisture content. This correlation could be explained by the state of the palm fruits used and the storage duration of these fruits after harvest (Ali et al., 2018).

The second group is composed of cottonseed oil which is strongly correlated with moisture, peroxide value, traces of soap, and mineral oil. These parameters are quality parameters of an oil. All these parameters, outside the acid value, are positively correlated on the F1 axis. The presence of traces of soap and mineral oil could come from a lack of control of the refining process or contamination of the seeds by mineral oil. These parameters attest that these oils are of lower quality. The peroxide rate attests to the degree of oxidation of the cottonseed oil. It would be linked to photo‐oxidation, thermo‐oxidation, and/or auto‐oxidation (Simo et al., 2019). The moisture content would be dependent on the quality of the equipment used, particularly clarifiers and dehydrators (Ahouansou & Agbobatinkpo, 2018).

These oils are therefore potentially harmful, combining all three parameters, that is, the peroxide value, traces of soap, and mineral oils (Gharbi et al., 2016). It should also be noted that this positive correlation between the acid and peroxide values could be attributed to the oxidation of the fatty acids liberated. The oxidation of the oil into unstable peroxides and hydroperoxides starts at the harvest of the seeds and continues during storage and processing (Motri et al., 2017).

## 5 CONCLUSION

From this study, it can be noted that the majority of the analyzed oil samples presented acceptable physicochemical parameters. However, trace amounts of soap, acid value, peroxide value, moisture, volatile matter, and mineral oil in some samples are a public health concern. In order to ensure the quality of the oils sold in the different cities, it would be essential for the authorities to set up competent structures in each locality for continuous quality control of the oils in order to preserve the health of the population. In addition, it would be necessary to raise awareness among producers and retailers of the extraction, storage and conservation processes that influence the stability of oils.

## CONFLICT OF INTERESTS

The authors have not declared any conflict of interests.

## Data Availability

The data that supports the findings of this study are available in the supplementary material of this article.
